# Detection of a Single Identical Cytomegalovirus (CMV) Strain in Recently Seroconverted Young Women

**DOI:** 10.1371/journal.pone.0015949

**Published:** 2011-01-10

**Authors:** Suchetha Murthy, Gary S. Hayward, Sarah Wheelan, Michael S. Forman, Jin-Hyun Ahn, Robert F. Pass, Ravit Arav-Boger

**Affiliations:** 1 Division of Infectious Diseases, Department of Pediatrics, Johns Hopkins Medical Institutions, Baltimore, Maryland, United States of America; 2 Sidney-Kimmel Cancer Research Center, Johns Hopkins Medical Institutions, Baltimore, Maryland, United States of America; 3 Department of Oncology, Johns Hopkins Medical Institutions, Baltimore, Maryland, United States of America; 4 Department of Pathology, Johns Hopkins Medical Institutions, Baltimore, Maryland, United States of America; 5 Department of Molecular Cell Biology, Sungkyunkwan University School of Medicine, Suwon, Korea; 6 Department of Pediatrics, University of Alabama at Birmingham, Birmingham, Alabama, United States of America; Yale Medical School, United States of America

## Abstract

**Background:**

Infection with multiple CMV strains is common in immunocompromised hosts, but its occurrence in normal hosts has not been well-studied.

**Methods:**

We analyzed CMV strains longitudinally in women who acquired CMV while enrolled in a CMV glycoprotein B (gB) vaccine trial. Sequencing of four variable genes was performed in samples collected from seroconversion and up to 34 months thereafter.

**Results:**

199 cultured isolates from 53 women and 65 original fluids from a subset of 19 women were sequenced. 51 women were infected with one strain each without evidence for genetic drift; only two women shed multiple strains. Genetic variability among strains increased with the number of sequenced genetic loci. Nevertheless, 13 of 53 women proved to be infected with an identical CMV strain based on sequencing at all four variable genes. CMV vaccine did not alter the degree of genetic diversity amongst strains.

**Conclusions:**

Primary CMV infection in healthy women nearly always involves shedding of one strain that remains stable over time. Immunization with CMVgB-1 vaccine strain is not selective against specific strains. Although 75% of women harbored their unique strain, or a strain shared with only one other woman, 25% shared a single common strain, suggesting that this predominant strain with a particular combination of genetic loci is advantageous in this large urban area.

## Introduction

Genetic variability among human cytomegalovirus (CMV) isolates is a well-known feature that has been used to link viral isolates epidemiologically and to determine the presence of secondary/new CMV infections. Molecular studies of infected infants and their mothers, children in day care centers, and organ transplant patients led to the conclusion that CMV isolates that were identical had a common source and that recovery of different strains from the same person implied infection with a secondary/new CMV strain [1]–[5]. Sequencing studies of CMV isolates have usually been limited to one or several genes often with the aim of identifying genotypes associated with virulence properties in immunocompromised patients or infants with congenital infection [6]–[8]. These studies provided information on genetic polymorphisms and geographical distribution of CMV genes, and the presence of secondary/new CMV strains. Most of these studies were based on genotyping cultured isolates of CMV from cross-sectional, convenience samples with limited or no longitudinal study and uncertainty as to when the study subjects acquired CMV.

While infection with multiple CMV strains is frequently reported in immunocompromised hosts, data in immune competent individuals is more limited. A key question in defining infection with a new CMV strain is whether multiple strains are acquired simultaneously at the time of initial infection or whether a single CMV strain is involved. To address this question, a cohort of healthy seronegative women who participated in a CMV glycoprotein B (gB) vaccine clinical trial were followed until they acquired CMV and for up to 34 months afterwards. CMV genotyping was based on direct PCR sequencing at several distinct gene loci including UL55, UL144, UL146 and UL09 from different body fluids collected at intervals after infection. In addition, it was possible to compare strains sequenced directly from body fluids to those sequenced after cell culture isolation, to address whether growth in culture might select for strains that grow more efficiently and therefore mask the detection of multiple strains.

## Results

### Definitions

#### Strain

Overall description of viral genomic structure based on the sum of all loci tested. Strains can be distinguished from one another by differences in one locus or multiple loci.

#### Genotype

A combination of subtype designations based on sequence data obtained from two or more gene loci.

#### Subtype

Cluster pattern based on sequence data at any one gene locus.

#### Variant

Minor nucleotide changes observed within a subtype.

#### Isolate

CMV recovered from a human specimen and passaged in culture a limited number of times.

### Demographics and samples

**Figure 1:**
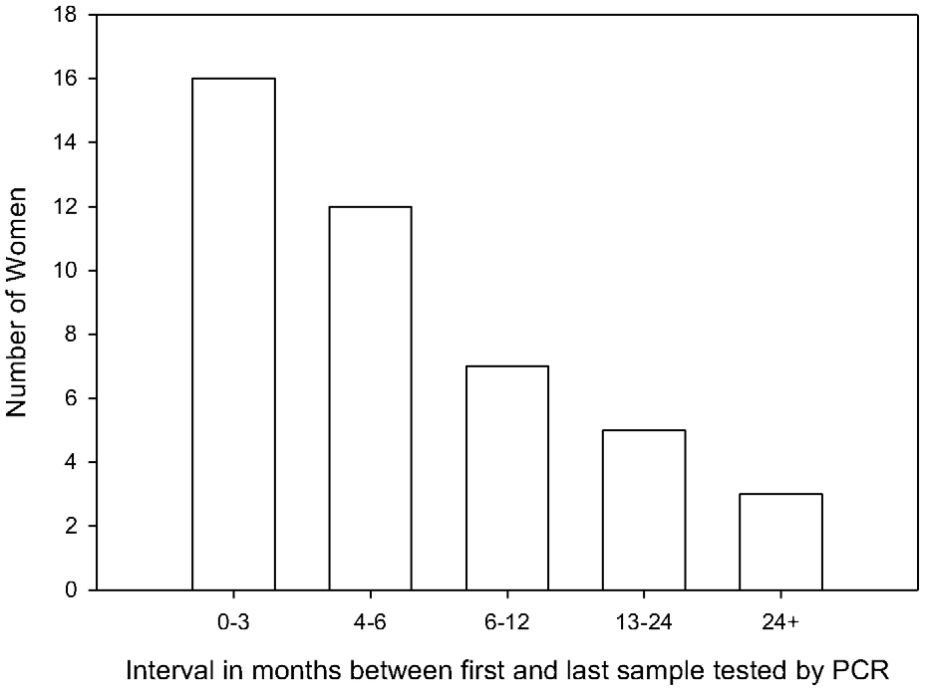
Interval (in months) between first and last sample tested by PCR. Samples available for PCR sequencing were collected from CMV-infected women at different times following seroconversion.

The study population was from the Birmingham, Alabama metropolitan area. The mean age of subjects was 19.6 years (range 15.3–33.9); 83% were African American and 17% were white, non-Hispanic. The time of infection was estimated to be the midpoint of the interval during which seroconversion was detected. All immunized women except one developed gB1 antibodies and neutralizing antibodies. CMV-infected women who received placebo had all developed gB1 antibodies. The median interval from time of infection to first viral cultures was 3 months. Cultures of specimens obtained within three months of infection were available in 50 women. In 3 women cultures were available only 6mo-18mo following seroconversion. Samples were available for PCR sequencing during follow-up of up to 34 months in 43 women. The average time between the first and last sample available for PCR sequencing was 10 months (Fig. 1).

A total of 199 tissue culture isolates of CMV from urine, saliva and vaginal fluid, from 53 women (range 1–13, average 3.8/participant; table 1) and 65 original fluids including urine, blood, vaginal and oral specimens from 19 women (range 1–7, average 3.4/participant) were analyzed. Of 53 women, 23 provided more than 4 isolates over time. These isolates always had an identical strain in the same woman. In cases where more than 3 samples were tested over time (n = 32, table 2) at least one body site was available for sequential testing.

**Table 1:** Number and source of CMV isolates from 53 CMV-infected women.

Number of Subjects	Number of isolates per subject	Source of isolates
10	1	7 urine, 2 saliva, 1 vaginal
11	2	13 urine, 5 saliva, 4 vaginal
9	3	17 urine, 6 saliva, 4 vaginal
23	≥4	66 urine, 47 saliva, 27 vaginal

**Table 2:** CMV strains detected in 53 CMV-infected women.

**1/B1/G1**	1/B1/G13	**1/B1/G7**	1/B2/ND	1*/A/ND
1*/B4/G5	1*/B4/G5	1*/B4/G7	1*/B6/ND	1**/A/ND
1**/A1C1/ND	1**/B1/ND	1**/B3/G11	1**/B3/G11	1**/B3/G11*
1**/B3/G11*	1**/B4/ND	2/A1C1/ND	2/A2/ND	2/B1/G13
2/B1/G9	**2/B3/ND**	2/B4/G13	**2/B4/G7**	**2/B4/G7**
**2/C/ND**	3*/B4/ND	3/A1/G1	3/A1/G12	**3/A1C1/ND**
**3/B3/G8**	**3/B3/G8**	**3/B4/ND**	3/C1/G11	**3/C1/G13**
3/C1/G8*	4/A1/G13	4/A1/G14	4/B1/ND	4/B4/G7
4/B4/G9*	**5/B2/G9**	**5/B2/G9**	**5/B2/G9**	**5/B2/G9**
**5/B2/G9**	**5/B2/G9**	5/B2/G9	5/B2/G9	5/B2/G9
5/B2/G9	5/B2/G9	5/B2/G9	5/B2/G9	**5/C1/ND**

Strain designations are based on the combined UL55/UL144/UL146 genotype. The strains were ordered from 1 to 5 based on UL55 subtypes and variants. UL146 subtypes given here are based on Dolan's nomenclature [10]. Bold entries are strains from vaccine-recipients. ND represents cases where a strain could be resolved based on UL55/UL144 only and UL146 sequencing was not attempted.

### One strain vs. multiple strains

A single CMV strain was detected in each of 51 women based on UL55 and UL144 sequencing from two or more isolates separated in time of collection by at least one month. The interval between the earliest and last isolates tested ranged from 1 to 34 months (median, 8 months). Isolates collected at 2 to 4 different points in time were available from 41/51; 21/51 had isolates from >4 different time points. In each of these 51 women the same strain was present in each isolate tested with no evidence of acquisition of new strains or of sequence changes based on testing of multiple genetic loci as described below.

Two of 53 participants were infected with multiple strains. In one of them, saliva and urine were available only at 18 months after seroconversion. Two different strains were detected, one in each fluid. To assess whether the two strains might occur in both fluids with one being more abundant than the other and therefore not detected by direct sequencing, PCR products obtained from saliva and urine were cloned into plasmids, and 20 independent colonies were picked from each site for sequencing. There was no evidence for low abundance strains existing along the predominant strain based on sequencing of 20 colonies. The second participant with two strains provided oral fluid near seroconversion in which only one strain was detected. Twelve months later a new strain was found in three urine samples while the original oral strain was unchanged. Sequencing of 20 plasmid clones from these PCR products again did not reveal minor strains coexisting along with the major strain.

### Variability within specific genetic loci

Our definition of CMV subtypes and genotypes is based on an extensive multilocus analysis of independent genomes obtained from a wide range of different clinical sources and of geographically diverse human populations (Zong JC, Alcendor DJ, Arav-Boger R, and Hayward GS, unpublished data). For the present study we chose to analyze four particular variable gene loci: the most commonly studied UL55/gB, of which one subtype (gB-1) is the vaccine strain, the truncated TNFα receptor-like gene UL144 [9], and the alpha chemokine ligand UL146, the most variable of all CMV genes with 14 known subtypes [10], [11]. Finally, in 13 women we also examined UL09, encoding a moderately variable protein with eight subtypes that is located on the other side of the UL region from UL144 and UL146.

**Figure 2:**
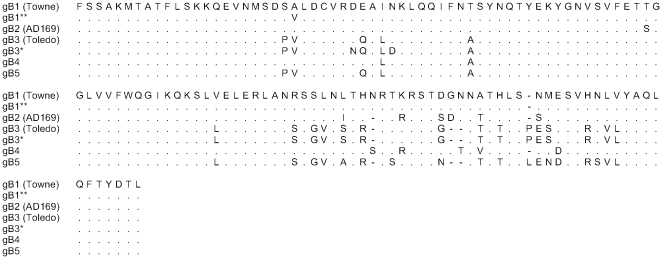
Alignment of gB subtypes. Amino acid alignment encompassing the proteolytic cleavage site of gB subtypes detected in the 53 CMV-infected women. Variants with amino acid changes within a subtype are depicted: gB1 (1 and 1**), and gB3 (3 and 3*). The gB1 prototype is Towne, gB2 prototype is AD169, and gB3 prototype is Toledo. The gB1* and the two variants of gB2 are associated with nucleotide changes only. Considering the five major gB subtypes, the two women with multiple strains had gB5/gB1, and gB4/gB1. The subtype distribution among the 51 women who shed one strain was as follows: 15 women shed gB1 (including variants), 9 women - gB2, 10 women - gB3, 4 women - gB4 and 13 women -gB5.

Five subtypes of gB were found among study participants with nucleotide polymorphisms between them ranging from 28 to124 bp. Subtype 1 contained three minor variants that differed from one another by 1, 4, and 5 nucleotides. Subtype 2 had two variants that differed by one nucleotide. Subtype 3 had two variants that had five nucleotide changes (Fig. 2 for amino acid alignment). There were no minor variants of subtypes 4 and 5. We judge the one nucleotide change to represent a genuine distinct variant rather than *Taq* polymerase error, because we have already identified this specific variant in other patient populations [4].

**Figure 3:**
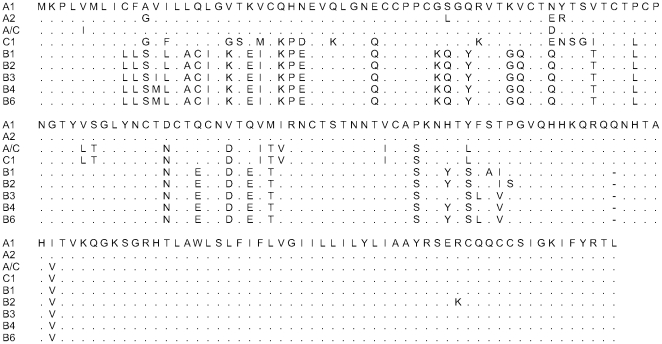
Alignment of UL144 subtypes. Amino acid alignment of full-length UL144 subtypes A, B, C, A/C. Among the 51 women who shed one viral strain the distribution of UL144 subtypes was: 6 women -UL144A, 7 women - UL144B1, 14 women - UL144B2, 6 women - UL144B3, 10 women - UL144B4, 1 woman - UL144B6, 3 women - UL144A1/C1 and 4 women - UL144C. The women with multiple strains had C/B3, and A1/B1. Please also refer to the phylogenetic tree, figure 4a.

**Figure 4:**
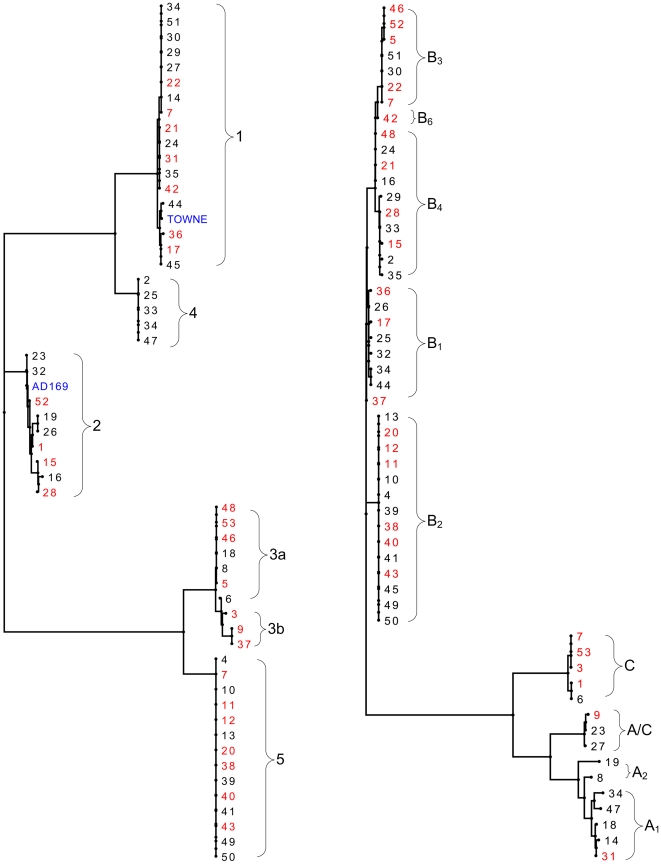
Phylogenetic tree of UL55 and UL144 subtypes. Left- UL55 DNA, right- UL144 DNA subtypes. Colored in red are -vaccine recipients, and in black- placebo recipients.

There were three UL144 subtypes (A, B, and C) and a recombinant group A/C (Fig. 3). Nucleotide changes between these subtypes ranged from 35–100 bp. There were five variants of UL144 subtype B (B1-4, and B6), with 1 to14 nucleotide changes between these variants. Subtype A included two variants, A1 and A2 that differed by 17 bp. Phylogenetic analysis of gB and UL144 DNA subtypes among vaccine and placebo recipients appear in Fig. 4.

**Figure 5:**
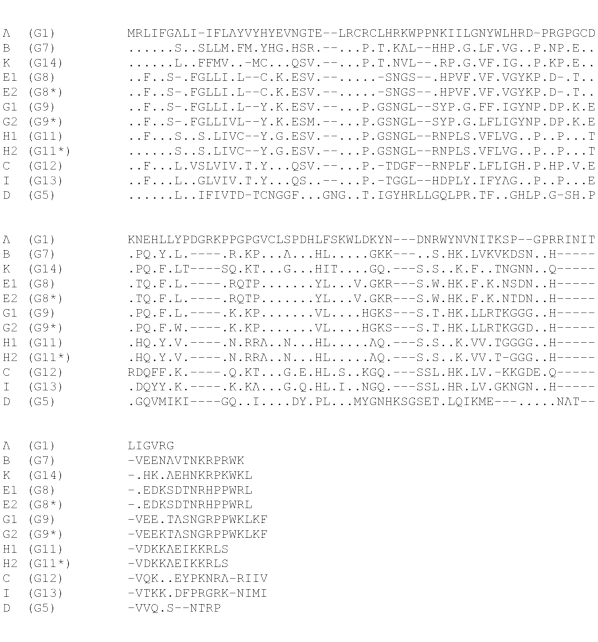
alignment of UL146 subtypes. Amino acid alignment of UL146 subtypes. Subtype definition by Dolan et al (10) appears in parentheses. Several variants in Dolan's subtypes G8, G9, and G11 have been detected in this cohort and are designated E1, E2, G1, G2, H1, and H2 based on common patterns of a small number of nucleotide and amino acid polymorphisms. Among 37 women the distribution of UL146 subtypes was: 14 women - G9, 1- G9*, 5 - G7, 5 - G13, 3 - G11, 2 - G11*, 2 - G8, 1- G8*, 2 - G5, 2 - G1, 1 - G12, and 1- G14. The women with multiple strains had G8*/G11, and G13/G14.

There were also nine highly diverged subtypes and three variants of UL146 (Fig. 5) similar to those seen in other studies [10], [12]. No nucleotide variations were observed amongst the viruses present in samples from different body fluids from the same participant. There was also no evidence for any subtype being found preferentially in a particular type of body fluid.

### Correlation between original fluids and cultured isolates

We sequenced UL55 and UL144 directly from original fluids and after propagation in cell culture from 19 participants in whom adequate original fluids were available. DNA sequence data obtained from cultured isolates matched the sequences obtained from original fluids.

### Minor CMV strains

There was no evidence for mixed infections in any samples examined by direct PCR sequencing. To further examine whether low abundance strains might exist but not observed by our population based sequencing, PCR products were cloned from 12 cultured isolates and from 10 original fluids, and 10 to 20 colonies each were picked for sequencing. In all cases, the parent strain obtained from direct sequencing of the PCR product was also found in sequences of all the cloned plasmids, with no evidence for any minor strains being present. Thus we judge that any low abundance strains that might be present were below a level of 5 to10% of the abundance of the major strain.

### Clonal infection

**Figure 6:**
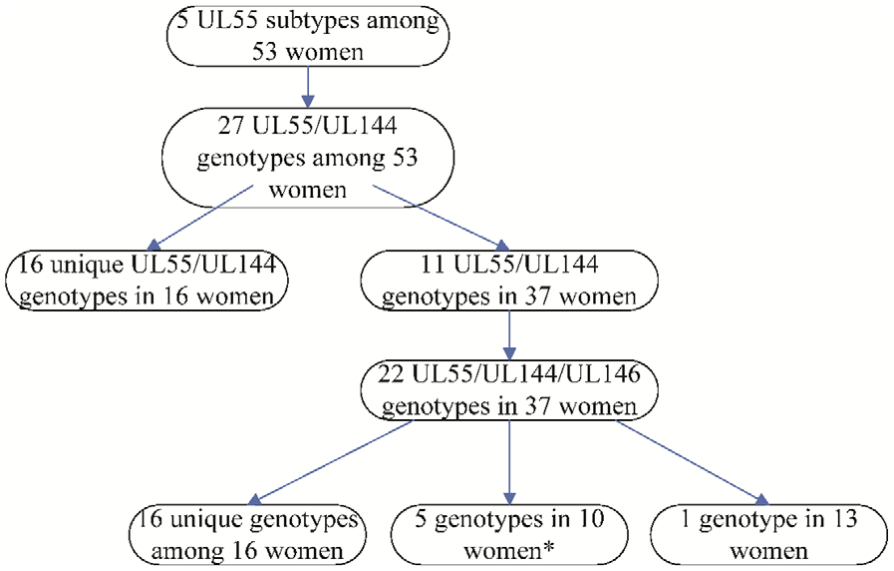
Breakdown of strain diversity in the study cohort based on sequencing of three hypervariable CMV genes. * The two women with two strains each had one unique strain (based on UL55/UL144) and one shared strain.

The number of distinguishable CMV strains detected in this study increased with the number of sequence loci analyzed. To resolve situations in which multiple subjects appeared to share the same strain additional genetic loci were sequenced. Based on UL55 alone, only 5 subtypes were found amongst the 53 women, but combining UL55 and UL144 subtypes yielded 27 genotypes (Fig. 6, table 2). At this level of analysis, 16 women were infected with a distinct UL55/UL144 genotype that was different from all other genotypes, whereas 37 participants fell into one of 11 different genotypes; each genotype was detected in 3 or 4 women, but one genotype was noticed in 13 women (Fig. 6, table 2).

To further resolve these 11 genotypes shared between the 37 participants we also sequenced the hypervariable UL146 gene locus. Adding the UL146 data revealed a total of 22 different genotypes; 16 women with identical UL55/UL144 genotype each had different unique UL146 subtypes. There were five pairs of women in which each pair shared a common UL146 sequence that was different from any of the other pairs (Fig. 6, table 2), as well as 13 women with the same UL55/UL144 who still shared exactly the same identical UL146 sequence. Therefore, the total number of women with their own unique strain based on triple loci UL55/UL144/UL146 sequencing was 32, whereas ten women shared five paired strains, and yet another 13 women were infected with the same strain based on sequencing of the UL55/UL144/UL146 loci (genotype 5/B2/G9). Furthermore, sequencing of the UL09 locus from these 13 women again yielded exactly the same subtype UL09-C with identical nucleotide sequences in all.

### Confirmation of sequencing data in a second laboratory

To completely rule out any potential source of artefactual laboratory contamination within or among the samples from these 13 participants who shared the same virus, we sequenced UL55 and UL144 from original fluids of 11 of the 13 participants. In all cases the results matched those obtained from cultured isolates. In addition, seven coded cultured isolates were sent directly from Alabama to a laboratory in Korea (Ahn JH) for independent DNA extraction, PCR and sequencing. Six of these samples were among the 13 women who shared the same CMV strain and one sample was from a woman infected with a different strain. After uncoding, the resulting genotypes again completely matched the data obtained in our Baltimore laboratory.

### Comparison of CMV strains in vaccine and placebo recipients

A high level of strain diversity was observed among both vaccine and placebo recipients. While some strains were shared between the two groups, others appeared only in the vaccine or in the placebo group (Fig. 5a, b, Table 2). The dominant strain 5/B2/G9/C, shared by 13 participants, was equally distributed among vaccine and placebo recipients. Among the two participants with multiple strains one received vaccine and the other received placebo.

## Discussion

We have shown that acquisition of multiple CMV strains at the time of primary infection is rare in healthy young women. Within the short period after primary CMV infection, shedding of a single CMV strain appeared to be the rule, and the same strain was detected from multiple different body sources in each participant during subsequent months. Viral strains analyzed by direct PCR sequencing in original fluids completely matched strains detected after propagation in cell culture. Surprisingly, although most women had their own unique strain, 25% of women enrolled in the study at different times and with no known epidemiologic links shared exactly the same CMV strain based on sequencing of four variable genetic loci.

In contrast to our findings in these recently infected healthy women, multiple CMV strains have been detected in AIDS patients [13], and transplant recipients [3], [14]. Previously CMV-seropositive transplant recipients became infected with new CMV strains from their donors [14]. Multiple CMV strains were detected in blood from 5 of 11 CMV-infected renal transplant patients [15]. In that study, all five patients were seronegative prior to transplantation, suggesting that normal seropositive donors may harbor and transmit multiple CMV strains or that the transplant recipients were susceptible to multiple exposures. In addition to acquisition of new strains over time, replacement of strains can also occur [16]. Immunocompromised hosts can become a reservoir for generation of new CMV strains *in-vivo* through recombination [17].

In all cases except two the same virus strain was observed in all body fluids available from the same participant. These two participants had two different strains each, but each strain was detected in a specific body fluid. Because of limited sample availability from these two women, we are unable to determine whether infection with both strains might have occurred around seroconversion or that a second exposure occurred later than the first. Compartmentalization of strains has been reported in transplant recipients [3], in HIV populations [18], [19], and a small series of women with STDs [20]. Based on our findings in the two women with multiple strains, the detection of a different virus in one or more body sites suggests that a new strain was occasionally able to overgrow the existing one so that the original one became undetectable.

Multiple CMV strains have been reported far less commonly in normal hosts and usually when repeated exposures to new sources of CMV would be expected. For example, four of eight women attending a sexually transmitted disease (STD) clinic excreted distinct CMV strains simultaneously at different sites or shed new strains when studied serially [20]. Infection with new CMV strains has also been reported in children attending day care centers [1], [21]. Previously immune mothers may acquire a new CMV strain during pregnancy and transmit it to their offspring [22]. We have reported on the detection of multiple CMV strains in autopsy tissues after in-utero death as a result of disseminated CMV infection [4]. One recent report on strain diversity in healthy CMV-seropositive women suggested that 15/16 women who tested PCR positive (total 2.9% PCR positivity in that cohort) were infected with more than one strain based on genotyping glycoproteins gN and gB from blood or urine samples [2]. However, the time of primary infection was unknown in these women, so it is not possible to draw conclusions as to diversity or homogeneity of CMV strains acquired around seroconversion. In addition, the detection of multiple genotypes from blood samples in that cohort may suggest that this specific population was at risk for infection with multiple strains. However, information on potential risks or immunodeficiencies was not available.

The importance of sequencing CMV genomes from original fluids stems from knowledge in CMV biology that clinical isolates undergo genetic changes and selection during passage in cell culture [23], and that initial load may determine growth rate. In our study there was no mismatch between strains sequenced from original fluids and strains sequenced from cultured isolates. Since we did not find multiple strains in original fluids we were unable to determine whether culture may select for strains that grow more efficiently.

Surprisingly, we found a completely identical CMV strain in 13 of 53 women who had no obvious epidemiological links. This result was based on sequencing at four widely spaced and variable loci (UL55/UL144/UL146/UL09). While identity occurs when comparing different viral strains at one or even two loci, multi-locus identity between four variable genes has never been reported between unrelated individuals. Although we cannot completely rule out the possibility that the single strain shared between the 13 women may prove to be distinct if sequence data from other genes becomes available, this seems extremely unlikely based on the known patterns of genetic variability within the chosen genes.

Considering the number of different possible subtypes at each genetic locus and assuming these subtypes are randomly distributed in the population, the probability that 13 of 53 women will be shedding the same strain is remote, unless a common source or exposure has been involved. Therefore, the wide occurrence of a single genetically identical strain amongst isolates across our study suggests that specific combinations of genotypes are not randomly distributed and may be detected more commonly within a constrained geographical area and time frame. We note that the same strain (5/B2/G9) was already detected nearly a decade ago in 2 of 21 neonates born in the Birmingham Metropolitan area with congenital CMV disease, AMS3 and SUR9 [4]. Upon additional sequencing these two strains also proved to be identical to each other at the nucleotide level over an additional 8,000-bp encompassing nine more highly variable genes RL05, RL06, RL12, RL13, UL01, UL37ex3, UL73, UL74 and UL120, as well as being identical to each other and to the 13 from the current study in UL09 (Hayward GS and Arav-Boger R, unpublished data). An Italian study that investigated transmission of CMV strains in 13 pregnant women and their families revealed that the detection of a single strain in original fluids or viral isolates was the rule, in agreement with our findings [24]. In each family, an identical strain was found based on UL55/UL144/UL146. Although strains from different families were usually distinct from one another, when comparing data from uni-locus sequencing, identical UL146, UL144 or UL55 DNA were observed sporadically among unrelated subjects.

The data presented here is based on PCR sequencing of viruses that were shed in different body fluids. We cannot completely exclude the possibility that some women were infected over time with new strains that were not shed. Type-specific serological methods (for gB and gH) are currently being developed [25], [26], and once their sensitivity and specificity is validated, they could be used to address this question in other more appropriate cohorts.

We conclude from this comprehensive sequencing project that: 1. Primary CMV infection in healthy young women predominantly involves one CMV strain as detected in both cultured isolates and original fluids. 2. Each strain remains stable at least during the first 2.5 years, albeit sampling only 1–2% of the CMV genome. 3. Shedding of multiple strains is evidently rare in the normal host over a period of several years. 4. Immunization with a CMV glycoprotein B subunit vaccine did not alter the distribution of CMV strains, and did not select for infection with particular viruses. 5. An unexpected finding was that a subgroup of women were infected with the same virus based on sequencing of four widely distanced genetic loci, suggesting that a viral strain containing specific combinations of variable genes may be advantageous and occur more commonly than other viral strains with different genetic makeup.

## Methods

### Study population

The study population was comprised of 53 women who acquired CMV infection while participating in a phase 2, randomized, double-blind, placebo controlled clinical trial of a CMV vaccine [27]. Clinical trial participants were CMV seronegative, healthy young women. They were immunized with CMV gB (based on Towne, gB-1 subtype) vaccine (Sanofi Pasteur, Marcy L'Etoile, France) with MF59 adjuvant (Novartis, Boston, MA) on a 0, 1 and 6 month schedule and were screened for CMV infection every three months for 3.5 years using an antibody assay which detects seroconversion to CMV proteins other than gB [28]. Institutional review board approval was obtained from University of Alabama at Birmingham and Johns Hopkins Hospital and all subjects signed an approved consent form.

### Viral isolation

Subjects with serologic evidence of infection were tested for CMV in blood, urine, saliva and vaginal swab from one month to 3.5 years after detection of infection. Aliquots of each specimen were stored at −80°C. Fresh urine, saliva and vaginal swab specimens were inoculated into cultures of MRC-5 cells (ATCC) or locally prepared human foreskin fibroblasts. Cultures were checked weekly for 4–6 weeks after inoculation; CMV was identified by its characteristic cytopathic effect. Tissue culture with CMV (primary isolate or first passage) was frozen at −80°C.

### DNA extraction and PCR amplification

Total genomic DNA was extracted from infected cells using a capture-column kit (Qiagen, Valencia, CA). CMV DNA was extracted from 400 µL of original samples - blood, urine, saliva or vaginal swab - using the MagAttract virus mini M48 kit (Qiagen, Valencia, CA) on Biorobot M48. Initially, UL55 (gB) and UL144 were amplified from all cultured isolates and from available original fluids. To further define strain diversity in participants that appeared to share the same genotype based on the combined UL55/UL144 data, UL146 was also amplified from 45 of these women. Finally, UL09 was amplified from 13 women who shared the same genotype based on UL55/UL144/UL146 data.

Primers for PCR amplification were reported [4], [29] or appear in Table 3. The sizes of the amplified loci were: 400 bp encompassing the proteolytic cleavage site of UL55, and 531 bp, 480 bp, and 687 bp representing the full length UL144, UL146 and UL09 protein coding regions. The reaction mixture consisted of 45 µL supermix (Invitrogen, Carlsbad, CA) 1 µL DNA, and 1 µL of each primer (10 µM). PCR reactions were set up in a dedicated, non-CMV laboratory. PCR products were separated by agarose gel electrophoresis. Appropriate DNA fragments were excised, purified using QIA quick gel extraction kit (Qiagen) and eluted with nuclease free water. All samples were subjected to direct sequencing with the same primers used for PCR.

**Table 3:** primers used in sequencing study.

*Primer*	*Sequence*
UL144 forward	5′-CGTATTACAAACCGCGGAGAGGAT-3′
UL144 reverse	5′-CTCAGACACGGTTCCGTAAAGTC-3′
UL144 nested forward	5′-CTTCCGGTAGGAGGCATGAAG-3′
UL144 nested reverse	5′-GACTTCATCGTACCGTGATC-3′
UL146-147 forward	5′- GTCATGGACGCAGTTTTG-3′
UL146-147 reverse	5′- GAACGATCTCGTCCGGTTC-3′
UL146 hemi-nested reverse 1	5′- CTAAAASATGGACGGCTAGG-3′
UL146 hemi-nested reverse 2	5′- GTCGTAATCTTCCARTTC-3′
UL55 forward	5′-TCCGAAGCCGAAGACTCGTA-3′
UL55 reverse	5′-GATGTAACCGCGCAACGTGT-3′
UL55 nested forward	5′-CATAGGTGAACTGCAGCTG-3′
UL55 nested reverse	5′-AGCATGGTGAAAAGAAGACG-3′
UL09 forward	5′-CATCTGTCTRCGAGCACCTC -3′
UL09 reverse	5′-GACCATCGGAAAAGATCATGG -3′
UL09 nested forward	5′-CAGTACGGACAAGTGTTYATG -3′
UL09 nested reverse	5′-GGTTCACGATATGGTTAATCAG -3′

### Cloning

In selected cases PCR products were also cloned and then sequenced. PCR reactions designated for cloning were obtained using high fidelity PCR supermix (Invitrogen, Carlsbad, CA). PCR products were cloned into Zero Blunt Topo (Invitrogen). Following ligation and transformation into E.coli cells, 20 recombinant colonies were picked and grown overnight at 37°C in LB broth containing 50 µg/mL kanamycin. Plasmid DNA was purified using High Pure Plasmid Isolation Kit (Roche, Pleasanton, CA), and plasmid inserts were sequenced with universal primers.

### Sequencing

Purified PCR products were sequenced using the BigDye Terminator Cycle Sequencing Kit (Perkin-Elmer Applied Biosystems, Foster City, CA). Sequencing products were analyzed with an ABI 3100 automated sequencer (Applied Biosystems). Nucleotide and amino acid sequences of clinical CMV strains and reference Towne, Toledo and AD169 strains were aligned using ClustalW [30] and MAFFT [31]. Phylogenetic trees were constructed using the neighbor- joining method in Mega4 [32].

### GenBank accession numbers

UL55- GU365817-GU365825, UL144- GU365826-GU365834, UL146- GU365835-GU365846, and UL09- HM542481.
